# Altered Expression of OsNLA1 Modulates Pi Accumulation in Rice (*Oryza sativa* L.) Plants

**DOI:** 10.3389/fpls.2017.00928

**Published:** 2017-06-02

**Authors:** Sihui Zhong, Kashif Mahmood, Yong-Mei Bi, Steven J. Rothstein, Kosala Ranathunge

**Affiliations:** ^1^Department of Molecular and Cellular Biology, University of Guelph, GuelphON, Canada; ^2^London Research and Development Centre, Agriculture and Agri-Food Canada, LondonON, Canada; ^3^The Samuel Roberts Noble Foundation, ArdmoreOK, United States; ^4^School of Biological Sciences, The University of Western Australia, CrawleyWA, Australia

**Keywords:** homeostasis, nitrogen, OsNLA1, permeability, phosphorous, toxicity

## Abstract

Current agricultural practices rely on heavy use of fertilizers for increased crop productivity. However, the problems associated with heavy fertilizer use, such as high cost and environmental pollution, require the development of crop species with increased nutrient use efficiency. In this study, by using transgenic approaches, we have revealed the critical role of OsNLA1 in phosphate (Pi) accumulation of rice plants. When grown under sufficient Pi and nitrate levels, OsNLA1 knockdown (*Osnla1-1, Osnla1-2*, and *Osnla1-3*) lines accumulated higher Pi content in their shoot tissues compared to wild-type, whereas, over-expression lines (OsNLA1-OE1, OsNLA1-OE2, and OsNLA1-OE3) accumulated the least levels of Pi. However, under high Pi levels, knockdown lines accumulated much higher Pi content compared to wild-type and exhibited Pi toxicity symptoms in the leaves. In contrast, the over-expression lines had 50–60% of the Pi content of wild-type and did not show such symptoms. When grown under limiting nitrate levels, OsNLA1 transgenic lines also displayed a similar pattern in Pi accumulation and Pi toxicity symptoms compared to wild-type suggesting an existence of cross-talk between nitrogen (N) and phosphorous (P), which is regulated by OsNLA1. The greater Pi accumulation in knockdown lines was a result of enhanced Pi uptake/permeability of roots compared to the wild-type. The cross-talk between N and P was found to be nitrate specific since the knockdown lines failed to over-accumulate Pi under low (sub-optimal) ammonium level. Moreover, OsNLA1 was also found to interact with OsPHO2, a known regulator of Pi homeostasis, in a Yeast Two-Hybrid (Y2H) assay. Taken together, these results show that OsNLA1 is involved in Pi homeostasis regulating Pi uptake and accumulation in rice plants and may provide an opportunity to enhance P use efficiency by manipulating nitrate supply in the soil.

## Introduction

High-yielding crop plants require large amounts of fertilizers that include essential macronutrients, such as P and N (Marschner, 1995). They are important for plant growth, development and productivity. P and N are taken up by plants mainly in the forms of inorganic phosphate and nitrate (Marschner, 1995). Unlike nitrate fertilizers, which can be produced from atmospheric N_2_, global P reserves are limited and projected to be a limiting factor for conventional agricultural production (Schachtman et al., 1998; FAO, 2008). From applied P fertilizer, only 15–30% is taken up by crops and the rest of it is lost to the environment or becomes unavailable due to the adsorption to soil particles, precipitation by other cations and conversion into organic forms by microbes (Marschner, 1995; Syers et al., 2008). The development of crops with increased P use efficiency may offer an opportunity to overcome the issues of high input costs and overuse of P fertilizers for sustainable agriculture. Better understanding of the molecular mechanisms and in-depth knowledge of the regulatory and signaling pathways may, therefore, be important for the genetic improvement of crops with increased P use efficiency.

P constitutes 0.2% of a plant’s dry weight (Schachtman et al., 1998) and is required in the execution of a wide range of structural and physiological processes such as membrane biogenesis, nucleic acid synthesis, energy transfer processes, regulation of enzymatic activities, and signal transduction (Theodorou and Plaxton, 1993; Marschner, 1995). Given the significance of P in the regulation of large number of physiological and developmental processes, plants maintain the cellular P homeostasis by employing a diverse range of molecular mechanisms to regulate Pi uptake, mobilization, and partitioning to various organs (Chiou and Lin, 2011). Plasma membrane-located Pi transporters (PHTs) regulate the initial uptake of Pi from the rhizosphere and subsequent Pi allocation inside plants (Chiou et al., 2001; Misson et al., 2004; Shin et al., 2004). In this regard, transcription factors such as PHOSPHATE STARVATION RESPONSE1 (PHR1), a MYB transcription factor, WRKY75, and ZAT6 have also been identified which modulate the expression of PHTs (Rubio et al., 2001; Devaiah et al., 2007a,b). Interestingly, PHR1 activity is itself modulated through protein sumoylation process by an E3 ligase, SIZ1, to regulate Pi starvation dependent responses in Arabidopsis (Miura et al., 2005). At the protein levels, PHOSPHATE TRANSPORTER TRAFFIC FACILITATOR1 (PHF1) facilitates the trafficking of high affinity Pi transporter PHT1.1 (Shin et al., 2004; Gonzalez et al., 2005). More recently, components of the 26S proteasome pathway, PHO2 (E2 ligase) and AtNLA (E3 ligase), have shown to play and important role in the regulation of P homeostasis in Arabidopsis (Aung et al., 2006; Chiou et al., 2006; Peng et al., 2007; Park et al., 2014). In addition, several members of the gene family containing the SPX domain are found to regulate Pi starvation response in various plant species, such as PHO1, AtSPX1, and AtSPX3 in *Arabidopsis thaliana* (Hamburger et al., 2002; Tian et al., 2007; Duan et al., 2008; Li et al., 2016), and OsSPX1, SPX2, SPX3, SPX4, SPX5 in rice (Wang et al., 2009, 2014; Lv et al., 2014; Shi et al., 2014).

We have previously reported the existence of crosstalk between nitrate and Pi in Arabidopsis (Kant et al., 2011). A detailed functional characterisation of AtNLA (NITROGEN LIMITATION ADAPTATION - an E3 ubiquitin ligase), PHO2 (PHOSPHATE2- ubiquitin conjugase) and a microRNA- miR827 revealed that Pi acquisition and homeostasis in Arabidopsis in greatly influenced by the availability of nitrate in soil (Kant et al., 2011; Lin et al., 2013; Nguyen et al., 2015). For example, when grown under either low nitrate or high Pi, both nla and pho2 mutants accumulated higher Pi levels in the leaves and showed Pi toxicity symptoms and early senescence. Over-expression of microRNA- miR827 in Arabidopsis that targets nla mRNA, also resulted in similar physiological responses (Kant et al., 2011). Interestingly, the ability to hyper accumulate Pi was not affected in nla or pho2 mutants when ammonium was used as sole nitrogen source, suggesting that Pi acquisition is independent of ammonium availability in the soil (Kant et al., 2011). In addition to the involvement in Pi homeostasis in plants, NLA is also responsible for source-to-sink remobilization of nitrate by mediating the degradation of NRT1.7 in Arabidopsis (Liu et al., 2017).

A protein BLAST analysis has shown the presence of AtNLA homolog in rice. However, its physiological significance has not been elucidated in the regulation of Pi homeostasis so far. Furthermore, information are lacking if the Pi acquisition and homeostasis in rice is altered by the presence of a certain type of N-source (nitrate or ammonium), with ammonium being the preferred N source for rice and the most abundant N source in the paddy fields (Ranathunge et al., 2016). In this study, we have analyzed the role of OsNLA1 in rice by growing rice OsNLA1 transgenic lines with different combinations and concentration levels of P (Pi) and N (nitrate or ammonium). Results showed that OsNLA1 negatively regulates Pi accumulation in rice. Pi acquisition was negatively affected by nitrate but not by ammonium concentration in the growth medium. Further, OsNLA1 interacted with rice-encoded PHO2 and successfully complemented the Arabidopsis *nla* mutant. These finding suggested a conserved nitrate-dependent role for NLA in Pi homeostasis in both monocots and dicots.

## Materials and Methods

### Identification of OsNLA1 Homolog, Phylogenetic Analysis, Generation and Characterization of Over-Expression and OsNLA1 Mutation of Rice Plant

The Japonica rice (*Oryza sativa* L.) variety of Nipponbare was selected for this study. To determine the presence of Arabidopsis NLA homolog in rice, its full-length protein sequence was BLAST searched in Rice Genome Annotation Database server^1^. The whole length of NLA protein sequences were used for phylogenetic analysis. The phylogenetic tree was computed with MEGA5 software^2^ using maximum likelihood method with 1000 bootstrap replicates.

The T-DNA line of *Tos17* insertion (NG3578) was obtained from rice the Tos17 insertion mutant database^3^. The genotyping of the T-DNA mutant plants was performed by using *OsNLA1*-specific primers NG3578LP (5′-GGCGACAGAGAAAATGCTTC-3′) and NG3578RP (5′-CTTGGCAAAATGGCATACCT-3′), along with *Tos17*-specific primer Tos17-tail6 (AGGTTGCAAGTTAGTTAAGA-3′). The construct for OsNLA1 over-expression was created by cloning the full-length OsNLA1 cDNA coding region into expression vector pCAMBIA 1300 driven by a maize ubiquitin promoter.

To generate the OsNLA1 –RNAi construct, a 430 bp OsNLA1 cDNA fragment was amplified by OsNLA1GLF (5′-CACCGAACAACGGTGCTATGGAGC-3′) and OsNLA1GLR (5′-TCACATGCCCAAGAATGCCCT-3′) then subcloned into the pENTR/D-TOPO cloning vector (Invitrogen, Carlsbad, CA, United States). Subsequently, the fragment was transferred inverted repeatedly into pANDA binary vector (miki 2004) downstream of a maize ubiquitin promoter.

All the transgenic lines were generated through *Agrobacterium*-mediated transformation. *Japonica* rice variety Nipponbare was used for all transgenic lines as well as Tos17 insertion line.

### Plant Growth in Hydroponics and Soil Pots for Experiments

To grow plants in hydroponics, rice seeds of different genotypes (WT, T-DNA or Osnla1-1, two RNAi lines or Osnla1-2 and Osnla1-3, and three over-expression lines or OsNLA1-OE1, OsNLA1-OE2, and OsNLA1-OE3) were germinated between moistened filter papers in Petri dishes for 7 days in a climatic chamber (day/night rhythm: 12/12 h, 28/23°C, light intensity: 550 μmol m^-2^ s^-1^). Seven days after germination, the seedlings were transferred to five different hydroponic systems for different nutrient treatments: (i) control (all nutrients were supplied at optimum levels as previously described by Ranathunge et al., 2003), (ii) low nitrate with 30 μM KNO_3_, (iii) low phosphate (LP) with 5 μM KH_2_PO_4_, (iv) high phosphate (HP) with 300 μM KH_2_PO_4_, and (v) low nitrate and high phosphate together. In treatments, all other nutrients were provided in an optimum concentration. The plants were grown in the same environmental conditions as those used for seed germination. Nutrient solutions were renewed every week. Four weeks old plants were used for genotyping, Pi and total P measurement of shoots and roots as well as nitrate and phosphate permeability measurements of roots.

In soil pot experiments, control plants were grown with full nutrients that included Sunshine LA4 (Sun Grow Horticulture, Agawam, MA, United States), peat moss and vermiculite (Perlite Canada Inc., Lachine, QC, Canada) with a ratio of 1:1:4, and 1 g of 14-14-14 Nutricote slow releasing fertilizer (Plant Products, Ltd Co, Brampton, ON, Canada). For P and N treatment experiments, the seedlings were planted in nutrient-free soil (peat-moss and vermiculite with a ratio of 1:4). It is known that positively charged nutrient ions can be bound to the soil particles, whereas, negatively charged ions can be lost by leaching (Marschner, 1995). Such bound and leached nutrient ions are not available for plants. Hence, soil-grown plants were provided with a greater concentration of nutrients than in hydroponics. The nutrient solution that had been used for hydroponics was modified with different KNO_3_ levels. To study how different nitrate levels affect/alter Pi uptake, a set of plants were grown in soil with low (3 mM) and optimum/sufficient (10 mM) KNO_3_ levels. For P treatment, plants were grown either with 0.1 mM (low) or 1 mM (optimum/sufficient) or 6.5 mM (high) KH_2_PO_4_ levels. Four weeks old plants were used for genotyping, phenotypic assays and other physiological and metabolic analyses.

In order to investigate whether or not ammonium affects Pi uptake in rice, wild-type, knockdown lines and over-expression lines were grown both in hydroponics and soil-pots with different ammonium levels but with an optimum/constant Pi level. In hydroponics, a concentration of 90 μM (NH_4_)_2_SO_4_ was used as low, whereas, 300 μM (NH_4_)_2_SO_4_ was used as the optimum. In soil-pot experiments, a relatively higher ammonium levels were used than in hydroponics due to binding of ions to negatively charged soil particles. In soil, the applied (NH_4_)_2_SO_4_ concentrations were 1 and 10 mM; the latter concentration being the optimum while the former concentration was sub-optimal. Four weeks old plants were harvested and used for Pi analysis.

### Expression Analysis of OsNLA1 Gene in Different Tissues of Wild-Type Plants

To analyze the expression level of OsNLA1 gene in different tissues of wild-type plants (leaf, root, stem, sheath, and inflorescence with open and closed spikelets), 2-month-old plants were harvested at noon and tissue samples were frozen immediately in liquid nitrogen. Total RNA was extracted from each tissue using TriZol regent (Invitrogen, Carlsbad, CA, United States) and RNEasy RNA extraction kit (Qiagen Inc., Toronto, ON, Canada). To eliminate genomic DNA from RNA, the samples were treated with RQ1 RNAse-free DNAse enzyme (Promega Cooperation, Madison, WI, United States). The cDNA was synthesized from total RNA with qScript cDNA SuperMix from Quanta Biosciences (Gaithersburg, MD, United States) and used for qRT-PCR analysis. The PCR primers were designed using the Applied Biosystems (Forster City, CA, United States) software Primer Express 2.0 (Supplementary Table S1). The qRT-PCR was carried out and analyzed with a 7300 real-time PCR system (Applied Biosystems, Foster City, CA, United States) according to the manufacturer’s instruction. For the selection of endogenous control (reference gene), *OsUBQ1, OsGAPDH*, and *OsActin8* were tested. *OsUBQ1* showed a uniform and most stable expression in all plant tissues as well as under different treatments. Hence, the constitutively expressed *OsUBQ1* was used as endogenous control. Three technical and three biological replicates were used for each measurement, and relative quantity was calculated using the 2^-ΔΔT^ method (Livak and Schmittgen, 2001).

### Expression Analysis of OsNLA1 Gene in Mutants and Over-Expression Lines

Quantitative RT-PCR was used to determine the expression level of *OsNLA1* in shoots of 4-week-old mutant and wild-type plants. Shoots from each plant were collected at noon and were frozen immediately in liquid nitrogen. Frozen samples were then ground into powder and used for RNA extraction. Preparation of cDNA and analysis of relative expression levels using qRT-PCR were performed as described earlier in the previous section. *OsNLA1* primers and endogenous control of *OsUBQ1* specific primers were used for this analysis. Three biological replicates were used for each assay.

### Analysis of Plant Phenotype and Measurement of Pi Content of Shoots

All plants (over-expression lines, RNAi lines and Tos17 insertion line along with wild-type) grown in hydroponics and soil with different nitrogen and phosphorous treatments (see plant growth section), were used for Pi analysis. In some experiments, total P in shoots and roots was also measured. After analyzing phenotype, plants were harvested and whole root and shoot tissues were homogenized with liquid nitrogen separately. Pi concentration was measured as previously described by Chiou et al. (2006), which was slightly modified from the method of Ames (1966). For analysis, approximately 0.1 g tissue powder was used for each sample. One milliliter of extraction buffer (1mM EDTA, 1 mM β–mercaptoethanol, 1 mM phenylmethylsulfonyl fluoride, 10 mM Tris and 100 mM NaCl) was added to the homogenized sample. 100 μl of supernatant was added to 900 μl of 1% glacial acetic acid and incubated at 42°C for 30 min. The samples were centrifuged for 5 min at 13,000 *g*. 300 μl of supernatant was mixed with 700 μl assay solution (0.35% NH_4_MoO_4_, 1.4% ascorbic acid, and 0.86N H_2_SO_4_) and incubated at 42°C for 30 min. The OD value of samples were measured at A820 using a spectrophotometer (MULTISKAN GO, Thermo Fisher Scientific, Vantaa, Finland). At least 3–5 biological replicates were used for each assay.

### Root Pressure Probe Measurements

The nitrate and Pi permeability of roots were measured by a root pressure probe. For this method, excised end segments of roots lacking laterals were used as previously described by Miyamoto et al. (2001). These measurements were performed for plants grown in both optimum (300 μM) and low (30 μM) nitrate levels for 3–4 weeks. Excised root segments were tightly but carefully connected to a root pressure probe without damaging them with the aid of cylindrical seals prepared from liquid silicone material (Xantopren; Bayer, Leverkusen, Germany). Once roots attained steady-state root pressures (usually it took 1–2 h), the osmotic experiments were performed. To measure nitrate and phosphate permeability of roots, the original nutrient solution was rapidly exchanged with a medium containing either 25 mM KNO_3_ (∼50 mOsmol/kg) or 25 mM KH_2_PO_4_ (∼50 mOsmol/kg), dissolved in the nutrient solution. During the osmotic experiment, the external root medium was rapidly stirred to mix the solution and to minimize the effects of the external, unstirred layers. The changes in root pressure in response to changes in the osmotic pressure of the medium were biphasic. There was a rapid water phase followed by a slower solute phase (see Miyamoto et al., 2001 for osmotic curves). From the solute phase, the permeability coefficients of KNO_3_ and KH_2_PO_4_ (*P*_sr_ in m s^-1^) were calculated according to Steudle et al. (1987):

(1) $$\begin{array}{c} k_{\mathrm{sr}}=\frac{\ln(2)}{t_{1/2}^{s}}=\frac{A_{r}\times P_{\mathrm{sr}}}{V_{x}} \end{array}$$

where *k*_sr_ is the rate constant of permeation of solutes. They were calculated from the half-time of solute exchange (*t*^s^_1/2_) of each osmolyte used here. *V*_x_ is the volume of mature xylem, which was 1 to 2% of the total root volume (Miyamoto et al., 2001).

Cutting experiments were conducted to validate the readings after each experiment. When the xylem of the root remained open, there was a quick drop in root pressure to zero and a drastic decrease in the *t*^w^_1/2_. If this did not occur, the results were discarded. All transport measurements were conducted at 27°C. Ten roots were used for each genotype with different nitrate levels.

### Complementation of Arabidopsis nla Mutant with Rice OsNLA1

The full-length coding sequence of OsNLA1 (Os07g0673200) was amplified using primers OsNLA-F (GGGGACAAGTTTGTACAAAAAAGCAGGCTTTATGAAGTTTGCCAAGAAGTACG), and OsNLA-R (GGGACCACTTTGTACAAGAAAGCTGGGTTTCACATGCCCAAGAATGC) and cloned into pDONR^TM^ 221 vector using a BP Clonase^TM^ enzyme mix (Invitrogen^®^). The coding region was then shuttled into compatible destination vector, pB7WG2D, using the LR Clonase^TM^ enzyme mix by following the instructions given in the manufacturer protocol (Invitrogen^®^, Invitrogen, Carlsbad, CA, United States). *Atnla* mutant was transformed with the destination vector using agrobacterium-mediated transformation by floral-dip method (Clough and Bent, 1998). Putative positive transformants were obtained by spraying with BASTA (0.1%). Integration and expression of the transgene OsNLA1 in Arabidopsis nla background was confirmed using PCR analysis in transgenic lines. Seeds from homozygous transgenic lines identified (from T2 generation) were used in *atnla* complementation assay. cDNA samples from five independent putative transgenic lines in nla background were subjected to PCR analysis to confirm the expression of OsNLA1 transgene. Primers set (qRTOsNLA1-F and qRTOsNLA1-R) was used to detect the amplification of OsNLA in Arabidopsis in different lines.

Wild-type (Col-0), at*nla* mutant and at*nla*-complementation lines transformed with OsNLA1, were grown under nitrogen sufficient (10 mM nitrate) and nitrogen limitation (3 mM nitrate) conditions as previously described by Peng et al. (2007). Rosette leaves from plants were harvested after 27 days of sowing, and Pi contents/concentrations were analyzed as described earlier by Chiou et al., 2006. Three to four replicates were used for each genotype.

### Yeast Two-Hybrid Assay

The full-length coding regions of the OsNLA1 and OsPHO2 (Phosphate over accumulator 2) were amplified using the primer pairs, yOsNLA1-F and yOsNLA1-R, and yOsPHO2-F and yOsPHO2-R, and cloned into pGEM-T easy vector. The coding regions of the OsNLA1 and OsPHO2 were then restricted from pGEM-T easy vector using Nde1 and BamH1, and ECOR1 and Xho1 restriction enzymes, and cloned into pGADT7-AD and pGBKT7-BD vectors, respectively. pGADT7-empty and pGADT7-OsNLA1constructs were transformed individually into Y187 strain. pGBKT7-empty and pGBKT7-OsPHO2 constructs were transformed into Y2HGold strain. Yeast mating between Y187 and Y2HGold strains was performed following the instructions given in Matchmaker Gold Yeast Two-Hybrid System User Manual (Clontech, United States). The hybrids were identified as positive clones based on their ability to grow DDO (SD/-Leu/-Trp) plates. Interactions were analyzed by growing the hybrids on DDO/X/A (SD/-Leu/-Trp/X-a-gal/Aureobasidin A) and QDO/X/A (SD/-Leu/-Trp/-His/-Ade/X-a-gal/Aureobasidin A) plates.

### Statistical Analysis

Data presented in the figures are means ± SD. Analysis of variance (ANOVA), and the LSD test were employed to compare the means of different genotypes with different nitrate and Pi levels at a confidence level of 95%.

## Results

### Phylogenetic Analysis of the NLA1 Homologs in Rice

**FIGURE 1:**
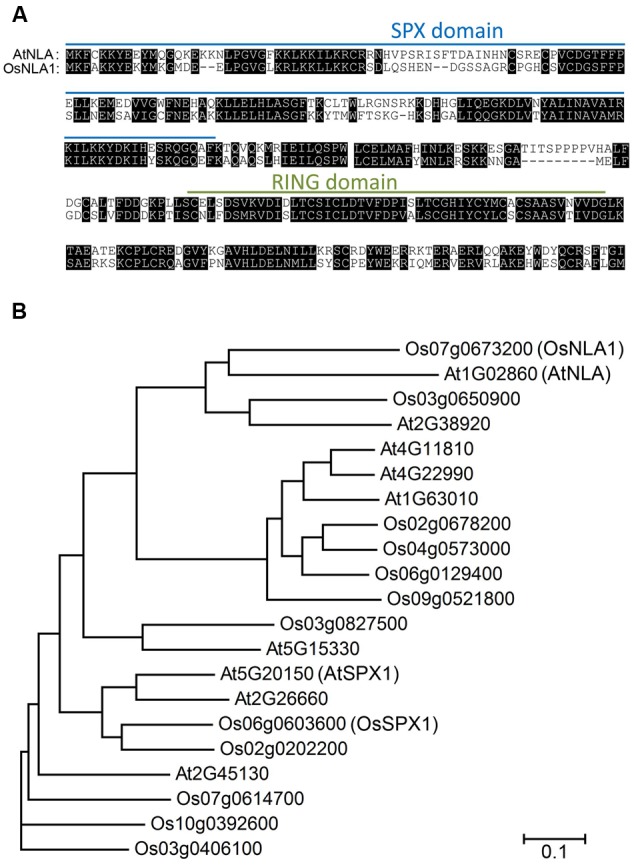
Comparison of AtNLA and OsNLA1 protein sequences and phylogenetic tree analysis. **(A)** The amino acid sequence of OsNLA1 has 59.7% sequence identity to AtNLA. OsNLA1 has an N-terminal SPX domain and a C-terminal RING domain. **(B)** Phylogenetic analysis with other related SPX proteins in Arabidopsis and rice.

Using the protein sequence of AtNLA gene (At1g02860), one homologous OsNLA1 in rice (Os07g0673200 or LOC_Os07g47590 given by Rice Annotation Project) was discovered by TBLASTN search^4^. The analysis of amino acid sequence of OsNLA1 showed that it has 59.7% sequence identity to AtNLA. OsNLA1 consisted of an N-terminal SPX domain and a C-terminal RING domain, which was similar to AtNLA (**Figure 1A**). Phylogenetic analysis with other related SPX proteins in Arabidopsis and rice showed that these genes together form a clade (**Figure 1B**).

### Expression Patterns of OsNLA1 in Different Tissues

**FIGURE 2:**
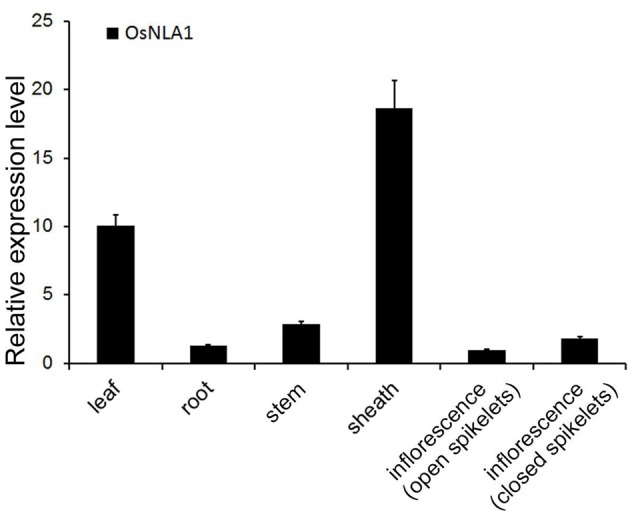
The relative expression of *OsNLA1* gene in different plant tissues. The expression of *OsNLA1* gene in leaf, root, stem, sheath, and inflorescence with open and closed spikelets was measured with qRT-PCR. The inflorescence with open spikelets was set as the reference or ‘1.’ The sheath and leaf tissues had the highest *OsNLA1* expression, whereas, roots and inflorescence had the least expression. Data are the means ± SD of three replicates (*n* = 3).

The spatial expression of *OsNLA1* was determined using quantitative RT-PCR analysis in different plant tissues (leaf, root, stem, sheath, and inflorescence with open and closed spikelets) of rice (Japonica rice variety of Nipponbare) (**Figure 2**). The tissue of open spikelets was set as the reference or 1 for comparison with other tissues. Although *OsNLA1* transcripts were detectable in all the tissue types examined, the sheath and leaf tissues had the greatest transcript levels, reaching approximately 18- and 10-fold higher than in the reference tissue, respectively, and 14- and 8-fold higher than in the roots, respectively (**Figure 2**). The lowest expression of *OsNLA1* was found to be in the open spikelets, which was approximately half of the inflorescence with closed spikelets (**Figure 2**).

### Decreased Levels of OsNLA1 Transcript Leads to an Increased Accumulation of Pi under High Phosphate Levels

**FIGURE 3:**
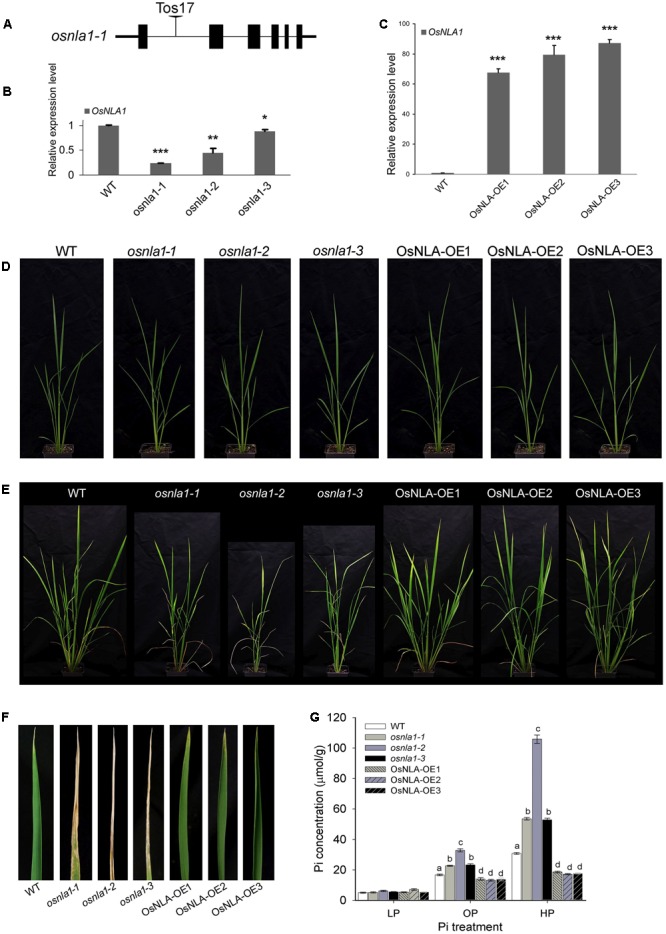
Analysis of phenotype of OsNLA1 transgenic lines under different Pi levels. **(A)** Selected homozygous Tos17 insertion line (NG3578) with T-DNA insertion in the first intron (*osnla1-1*), **(B)** the relative expression of OsNLA1 gene in T-DNA line (*osnla1-1*) and two independent RNAi lines (*osnla1-2* and *osnla1-3*), and **(C)** the relative expression of OsNLA1in three over-expression lines (OsNLA-OE1, OsNLA-OE2, and OsNLA-OE3). The comparison was made relative to the wild-type, and determined by qRT-PCR analysis. Phenotypic analysis of plants grown in different Pi levels for 4–5 weeks after sowing seeds: **(D)** low and **(E)** high. With high Pi, knockdown lines showed **(E)** shorter plants with reduced tiller numbers and **(F)** severe leaf necrosis. **(G)** Pi content of leaf tissues, harvested from plants grown with low Pi (LP), optimum Pi (OP), and high Pi (HP) levels. Significance levels of *P* < 0.05 or *P* < 0.01 or *P* < 0.001 are denoted by ^∗^ or ^∗∗^ or ^∗∗∗^, respectively. Different letters indicate significant differences at *P* ≤ 0.05 level (ANOVA, LSD test). Data are means ± SD of three to five replicates (*n* = 3–5).

To determine the role of OsNLA1 in rice (in Pi homeostasis), both loss-of-function and gain-of-function approaches were employed. In the context of the loss-of-function approach, a homozygous Tos17 insertion line (NG3578) with T-DNA insertion in the first intron was included in the study (**Figure 3A**). qRT-PCR analysis of the T-DNA line (referred to as *Osnla1-1*, henceforth) showed that the transcripts of OsNLA1 were still detectable (∼24% of the wild-type) suggesting that *Osnla1-1* is not a null mutant, and thus represent a knockdown mutation (**Figure 3B**). In addition to this, RNAi lines expressing an OsNLA1 hair-pin construct were also generated. Amongst these, two independent RNAi lines, *Osnla1-2* and *Osnla1-3*, which demonstrated the highest decrease in the transcript levels of the endogenous OsNLA1 compared to the wild-type, were selected for further analysis (**Figure 3B**). The TOS17 line (*Osnla1-1*) and RNAi lines (*Osnla1-2* and *Osnla1-3*) lines have the same background. In the case of the gain-of-function approach, transgenic lines overexpressing OsNLA1 under the UBQ promoter were generated. Three independent over-expression lines, OsNLA-OE1, OsNLA-OE2, OsNLA-OE3 with maximum expression levels of the OsNLA1 transgene were selected for further analysis (**Figure 3C**).

Alteration of OsNLA1 expression did not affect the shoot biomass content of rice plants under optimal nutrient levels (nitrate and Pi; **Supplementary Figure S1**). Even under low Pi (0.1 mM), all the transgenic lines and the wild-type grew similarly (**Figure 3D**). However, when grown under high phosphorus (HP) condition, the knockdown lines, *Osnla1-1, Osnla1-2*, and *Osnla1-3*, showed reduced plant size with lower tiller numbers and leaf necrosis (starting from the tip of the leaf blade), which were apparent Pi toxicity symptoms (**Figures 3E,F**). Biochemical analysis of the leaf tissues showed that knockdown lines accumulated significantly higher levels of Pi compared to wild-type, especially under HP level (**Figure 3G**). On average, under high Pi level, the knockdown lines had 1.4- to 3.8-fold greater Pi content compared to the wild-type. In contrast, the over-expression lines accumulated significantly lower levels of Pi containing 50–60% of the Pi content of wild-type (**Figure 3G**). Interestingly, even under the optimal Pi level, the transgenic lines exhibited the same trend for Pi accumulation but at a much lesser level. However, no differences were found for Pi accumulation when transgenic lines were grown under low Pi level (**Figure 3G**).

### OsNLA1 Negatively Modulates Pi/P Accumulation under Nitrate Limitation Conditions

**FIGURE 4:**
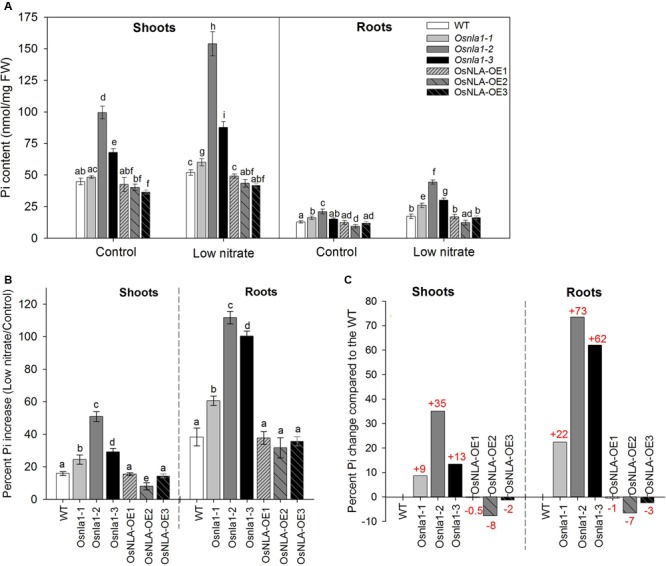
Analysis of Pi accumulation in shoots and roots of OsNLA1 transgenic lines under sufficient and low nitrate levels. **(A)** Pi content of shoots and roots of wild-type, knockdown and over-expression lines, grown with sufficient (Control) and low nitrate levels in hydroponics for 4 weeks. Knockdown lines (*osnla1-1, osnla1-2*, and *osnla1-*3) had the greatest Pi contents in shoots and roots, whereas, over-expression lines (OsNLA-OE1, OsNLA-OE2, and OsNLA-OE3) had the lowest. Low nitrate treatment markedly increased Pi content in both shoots and roots. **(B)** The percent Pi increase by low nitrate treatment compared to the control. **(C)** The percent Pi change of knockdown and over-expression lines by low nitrate treatment, relative to the wild-type. Different letters indicate significant differences at *P* ≤ 0.05 level (ANOVA, LSD test). Comparison was done for shoots and roots separately. Data are means ± SD of three to five replicates (*n* = 3–5).

In general, irrespective of the genotype, when grown under sufficient and low nitrate levels, shoots had markedly greater Pi content than roots (**Figure 4A**). In all genotypes, low nitrate treatment significantly increased Pi content of both leaf and root tissues. The comparison of genotypes showed that T-DNA and RNAi lines had significantly greater Pi contents in shoots and roots than wild-type at both growth conditions but it was more pronounced under low nitrate levels (**Figure 4A**). In contrast, the over-expression lines had slightly lower Pi contents than wild-type in both shoots and roots (**Figure 4A**). The calculated percent Pi increase under low nitrate revealed that there was a marked increment in both shoots and roots but this was more pronounced in the latter (**Figure 4B**). This data clearly showed that the knockdown plants had the greatest percent Pi increase over wild-type and over-expression lines (**Figure 4B**). The Pi increase was the same for wild-type and over-expression lines. When compared with the wild-type, the percent Pi increase of knockdown lines under low nitrate was 9–35% in shoots and 22–73% in roots (**Figure 4C**). This percent Pi change was slightly negative for over-expression lines. On average, it was 0.5–8% and 1–7% lower than wild-type for shoots and roots, respectively. In summary, the knockdown lines had the greatest Pi accumulation under low nitrate treatment, whereas over-expression lines had the lowest Pi accumulation.

In all genotypes, a low nitrate treatment led to a markedly increased total P content in both leaf and root tissues, which was the same as for Pi (**Supplementary Figure S2A**). The comparison of genotypes showed that OsNLA1 knockdown lines had greater total P contents in shoots and roots than wild-type and over-expression lines grown at a low nitrate level (**Supplementary Figure S2A**). The total P content of over-expression lines and wild-type was the same, except for roots grown under low nitrate. The percent P increase under low nitrate showed that there was a marked increment in both shoots and roots but the knockdown lines had the greatest values, whereas, over-expression lines had the lowest values compared to wild-type (**Supplementary Figure S2B**). On average, the knockdown lines had 15–62% total P increase in shoots and 15–52% in roots than that of wild-type (**Supplementary Figure S2C**). This percent P change was slightly negative for over-expression lines. On average, it was 1–4% and 5–8% lower than wild-type for shoots and roots, respectively.

### OsNLA1 Knockdown Lines Display Pi-Induced Toxicity Symptoms and Greater Pi Accumulation under Combined High Phosphate and Low Nitrate Treatments

**FIGURE 5:**
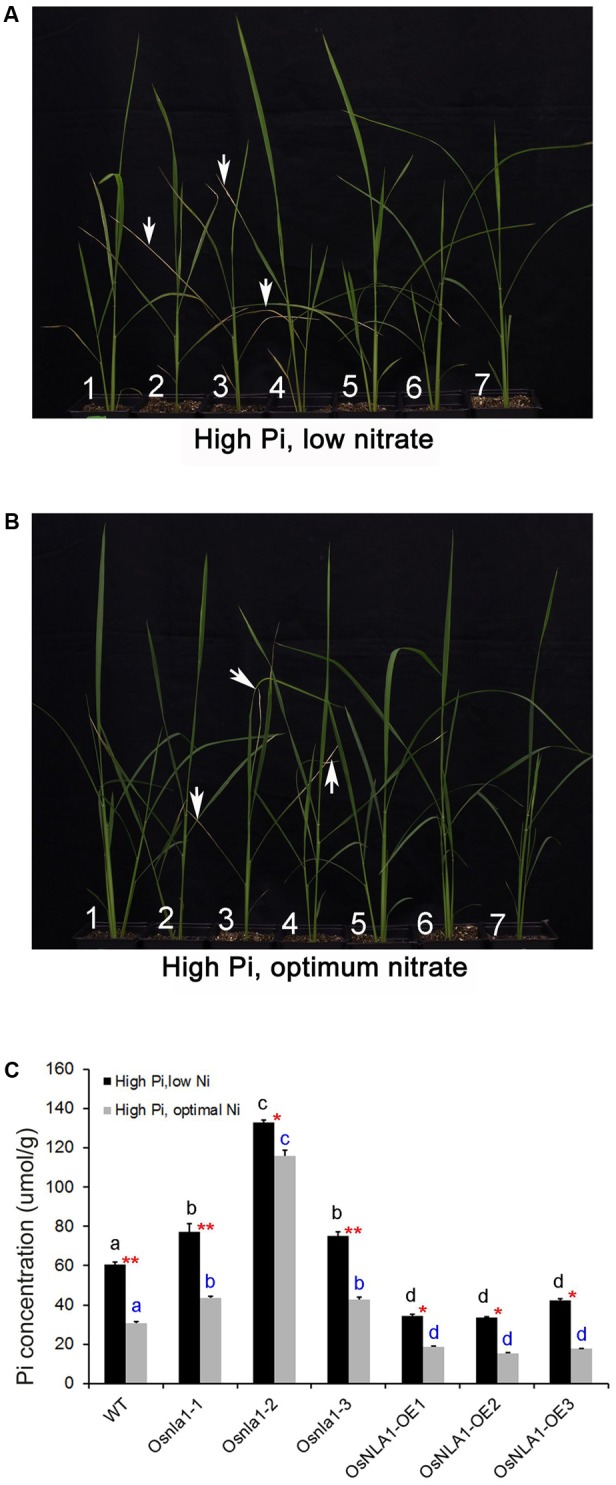
Pi accumulation and subsequent Pi toxicity symptoms of OsNLA1 transgenic lines under combined high phosphate and low nitrate treatments. Two sets of soil-grown plants that included **1**- wild-type, **2**- *osnla1-1*, **3**- *osnla1-2*, **4**- *osnla1-3*, **5**- OsNLA-OE1, **6**- OsNLA-OE2, and **7**- OsNLA-OE3 were grown in two different treatments with combination of Pi and nitrate together for 4 weeks. Knockdown lines showing chlorosis/necrosis patches at the tip of the leaf blade (arrows) under **(A)** combined high Pi and low nitrate, and **(B)** combined high Pi and optimum nitrate treatments. **(C)** Pi content of the shoot of plants; knockdown lines have the greatest Pi accumulation, whereas, over-expression lines have the least accumulation. For each genotype, low nitrate treatment significantly increased Pi concentration in shoots of plants (significance level of *P* ≤ 0.05 and *P* ≤ 0.01 denoted by ^∗^ and ^∗∗^, respectively; two sample *t*-test). Different letters indicate a significant difference at *P* ≤ 0.05 level between genotypes within a growth condition (ANOVA, LSD test). Data are means ± SD of three to five replicates (*n* = 3–5).

The resulted negative plant phenotypic effects, such as shorter plants and less tiller numbers of OsNLA1 knockdown plants were apparently due to the accumulation of high P/Pi levels under low nitrate or high phosphate levels (**Figures 3, 4**). These observations led us to test the response of transgenic lines grown in both low nitrate and high phosphate together. As expected, when grown in low nitrate and high phosphate levels together, plants showed a more severe leaf chlorotic and necrotic phenotype compared to the plants grown in optimum nitrate and high phosphate together (**Figures 5A** vs. **5B**). All knockdown lines had stronger phenotypes with leaf necrosis at the tip of the leaf blade than the wild-type grown in low nitrate and high phosphate together (**Figure 5A**). The phenotype of the over-expression lines and the wild-type was the same. These chlorotic/necrotic patches in knockdown lines were what one would expect for Pi toxicity symptoms due to hyper-accumulation of Pi in the shoot. Consistent with this hypothesis, in both growth conditions, the measured Pi content of shoots showed that the knockdown lines had markedly greater levels than wild-type, and over-expression lines (**Figure 5C**). The latter had the lowest Pi contents grown under either low or optimum nitrate levels with high Pi (**Figure 5C**). In all genotypes, low nitrate treatment significantly increased Pi acquisition in rice plants. In summary, OsNLA1 knockdown lines showed the strongest Pi toxicity symptoms and the greatest Pi accumulation under high Pi/low nitrate levels. In contrast, all three over-expression lines had the mildest leaf necrotic symptoms and the least Pi accumulation under high Pi/low nitrate levels.

### OsNLA1 Knockdown Lines Do Not Over-Accumulate Pi under Low Ammonium Level

To test whether there is a relationship between ammonium and Pi accumulation in rice plants, wild-type and *Osnla1-1* knockdown line were grown in both hydroponics and soil, providing them with optimum Pi and different ammonium levels (optimum and low). The results showed that there was no clear correlation between different ammonium levels and Pi accumulation in the shoot of wild-type and *Osnla1-1* knockdown line (**Supplementary Figure S3**). Lower ammonium treatments failed to accumulate higher Pi levels in the shoot of both genotypes either in soil or hydroponics, which was in contrast compared to plants grown at a low nitrate level (see **Figure 4A**). *Osnla1* had significantly greater Pi content in the shoot than wild-type in all ammonium treatments.

### Roots of OsNLA1 Knockdown Lines Are More Permeable to Pi under a Low Nitrate Level

**FIGURE 6:**
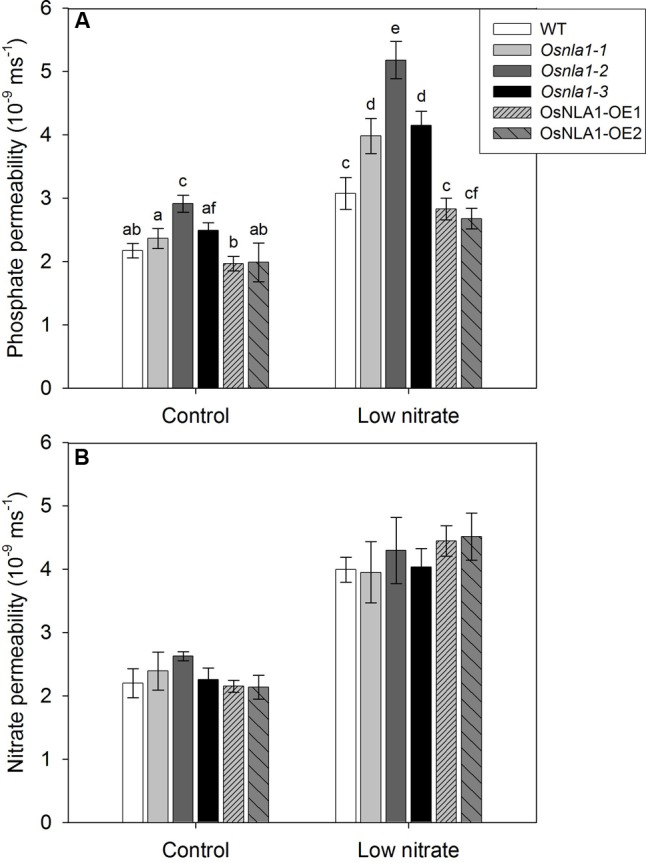
Permeability of roots (*Ps*_r_) for Pi and nitrate of OsNLA1 transgenic lines under low and optimum nitrate levels. The Pi and nitrate permeability was measured using a root pressure probe for adventitious roots of plants grown in hydroponics for 4 weeks in either optimum/control (300 μM), or low (30 μM) nitrate levels. **(A)** Comparison of Pi permeability of roots grown at optimum and low nitrate levels. In all genotypes, low nitrate treatment enhanced Pi permeability of roots, but more pronounced in knockdown lines (*osnla1-1, osnla1-2*, and *osnla1-3*). **(B)** Comparison of nitrate permeability of roots grown in different nitrate levels. All genotypes have the same permeability at each nitrate treatment. Plants grown in low nitrate had enhanced nitrate permeability of roots in all genotypes. Different letters indicate significant differences at *P* ≤ 0.05 level (ANOVA, LSD test). Data are means ± SD of ten replicates (*n* = 10).

The Pi and nitrate permeability (*Ps*_r_) was measured for adventitious roots grown in either optimum/control (300 μM), or low (30 μM) nitrate levels. In general, regardless of the genotype, roots grown in low nitrate had higher Pi and nitrate permeabilities than roots from the optimum nitrate treatment (**Figures 6A,B**). When grown and measured under low nitrate level, the Pi permeability of roots of the knockdown lines had markedly greater values than wild-type and the over-expression lines (**Figure 6A**). Under low nitrate level, on average, the knockdown lines had 34–66% greater permeability values than wild-type plants (*P* < 0.05; **Figure 6A**). In contrast, the *Ps*_r_ of roots for Pi was the same for wild-type and over-expression lines under both nitrate levels. Further, there was no significant difference in nitrate permeability of roots between the genotypes (**Figure 6B**). Plants grown in low nitrate had higher nitrate permeability, likely due to weak root barriers, which were induced by sub-optimal N that resulted in higher permeability. In summary, when plants were provided with low nitrate, it enhanced Pi uptake of roots but it was more pronounced in the knockdown lines than in the wild-type and over-expression lines.

### OsNLA1 Complements the Arabidopsis NLA Mutation

**FIGURE 7:**
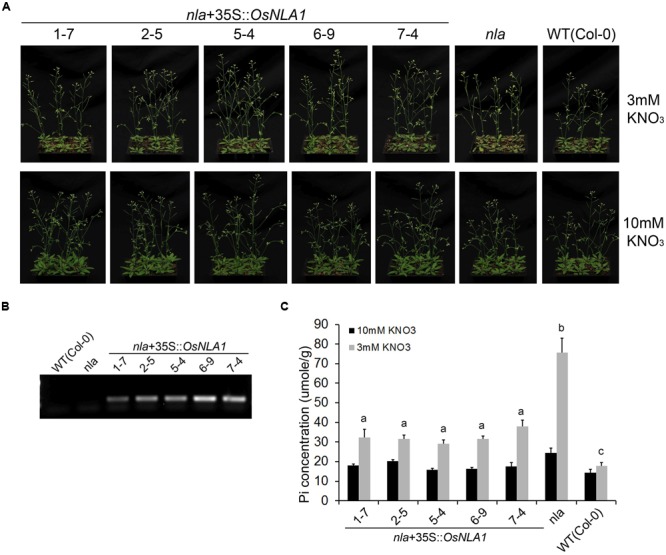
Complementation of Arabidopsis nla mutants by OsNLA1 and the content of Pi in plants grown at optimum and low nitrate level. **(A)** Five independent transgenic lines, expressing OsNLA1 under 35S CaMV promoter in Arabidopsis *nla* background; plants were grown under nitrate sufficient (10 mM KNO_3_) and limitation (3 mM KNO_3_) conditions for 27 days. Under nitrate sufficient conditions, all plants grew normally without obvious early senescence symptoms. Under nitrate limitation conditions, *nla* mutant displayed early senescence. **(B)** The expression of OsNLA1gene in complemented lines, *nla* mutants and wild-type plants, analyzed by RT-PCR. Complemented lines showed varied levels of the expression of OsNLA1 gene. **(C)** Pi content (μmole/g FW) in the shoot of plants grown with sufficient and low nitrate levels. Different letters indicate a significant difference at *P* ≤ 0.05 level between genotypes with low nitrate treatment (ANOVA, LSD test). Data are means ± SE of four replicates (*n* = 4).

Given the high sequence similarity between rice and Arabidopsis NLA proteins, we next examined if rice NLA could complement the *nla* mutation in Arabidopsis. Five independent transgenic lines, expressing rice NLA under control of the 35S CaMV promoter in the Arabidopsis *nla* background, were grown under nitrogen (N) sufficient (10 mM KNO_3_) and limitation (3 mM KNO_3_) conditions (**Figures 7A,B**). Under N sufficient conditions at 27 DAS, all the transgenic lines including *nla* and wild-type grew normally with no drastic senescence symptoms (**Figure 7A**). However, under N limitation conditions, the *nla* mutant displayed the premature senescence phenotype compared to wild-type (**Figure 7A**). Interestingly, all five transgenic lines expressing rice NLA did not show premature senescence and grew phenotypically similar to wild-type under N limitation condition. These observations were further confirmed through the analysis of Pi content. When grown under optimum N level, on average, the Pi content of the *nla* mutant was 1.7-fold greater than the wild-type, whereas, complemented lines had 1.4-fold of the wild-type (**Figure 7C**). In contrast, the over-accumulation of Pi was more pronounced under low N conditions in *nla* mutant reaching 4.3-fold compared to wild-type. However, the complemented lines had significantly lower content of Pi than the *nla* mutant with 1.6- to 2.1-times greater than the wild-type (**Figure 7C**). These results suggest that *OSNLA1* partially complemented the *nla* mutant phenotype.

### OsNLA1 Interacts with OsPHO2 *In Vitro*

**FIGURE 8:**
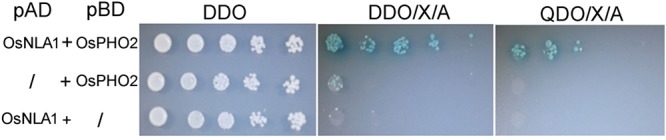
Analysis of interaction between OsNLA1 and OsPHO2 using yeast-two hybrid (Y2H) assay. Yeast hybrid cells carrying combination of pGAD7-AD and pGBKT7-BD constructs were spotted on DDO (selection for both plasmids), DDO/X/A (for relaxed selection) and QDO/X/A (for stringent selection). Yeast hybrid cells only carrying pGADT-OsNLA1 and PGBKT7-OsPHO2 combination were able to grown on DDO/X/A and QDO/X/A plates. {pAD - pGADT7-AD; pDB – pGBTK7-DB; DDO - SD/-Leu/-Trp/(double dropout); DDO/X/A (SD/-Leu/-Trp/X-a-gal/Aureobasidin A); QDO/X/A (SD/-Leu/-Trp/-His/-Ade/X-a-gal/Aureobasidin A)}.

A yeast two-hybrid (Y2H) assay was carried out to determine whether there is an interaction between OsNLA1 and OsPHO2 as earlier found for Arabidopsis (Park et al., 2014). In this assay, one protein is translationally fused with the DNA binding domain (GAL4-BD) and the other with the activation domain (GAL4-AD) of the GAL4 transcription factor. The result showed that there is an interaction between OsNLA1 and OsPHO2. The yeast hybrid cells carrying the pAD-empty and pBD-OsPHO2, and pAD-OsNLA1 and pBD-empty constructs were unable to grow on DDO/X/A and QDO/X/A plates (**Figure 8**). However, the hybrids harboring the pAD-OsNLA1 and pBD-OsPHO2 constructs were able to grow on DDO/X/A and QDO/X/A plates (**Figure 8**), suggesting that OsNLA1 and OsPHO2 interact with each other and this interaction may play an important role in phosphate acquisition/homeostasis of rice plants.

## Discussion

In previous studies, it has been shown that the Arabidopsis NITROGEN LIMITATION ADAPTATION (NLA) gene is involved in adaptive responses to low nitrogen conditions, where *nla* mutant plants displayed an abrupt early senescence (Peng et al., 2007, 2008). When grown with sufficient nitrogen, the *nla* mutant was indistinguishable from wild-type. The induction of premature senescence in the Arabidopsis *nla* mutant under nitrogen limitation condition has been demonstrated to be the result of over-accumulation of Pi to a toxic level, which was shown to be nitrate-dependent (Kant et al., 2011). Similar to the *nla* mutant, the Pi overaccumulator PHO2 mutant also showed Pi toxicity under low nitrogen levels (Kant et al., 2011). For the first time, we investigated the role of the rice NLA (OsNLA1) on Pi homeostasis in plants grown on different nitrate and ammonium levels. The new findings clearly demonstrated that OsNLA1 has an important role in regulating Pi homeostasis in rice plants in a nitrate dependent-manner while ammonium levels had no effect on Pi accumulation.

Phylogenetic analysis of SPX proteins in Arabidopsis and rice showed that SPX family members have either a singular SPX-domain or it is combined with a RING or MFS (major facility superfamily) or EXS domain (**Figure 1**). OsNLA1 was identified as AtNLA ortholog in rice, based on its high sequence similarity at the amino acid level (59.7%) and for possessing both the SPX and RING domains (**Figure 1B**; Peng et al., 2008). Conservation of functional similarity between OsNLA1 and AtNLA was further tested by OsNLA1’s ability to complement the *nla* mutation in Arabidopsis under nitrogen limitation conditions. Indeed, the toxicity symptoms and premature senescence associated with hyper accumulation of Pi were alleviated when OsNLA1 was expressed in the Arabidopsis *nla* background under nitrogen limitation conditions (**Figure 7**). Interestingly, several members from different clades of the SPX family have been implicated in maintaining Pi homeostasis in different plant species. For example, the SPX domain containing proteins such as PHO1, AtSPX1 and AtSPX3 in *A. thaliana* (Tian et al., 2007; Duan et al., 2008; Li et al., 2016), and OsSPX1, SPX2, SPX3, SPX4, SPX5 in rice, have been shown to play important roles in the regulation of Pi homeostasis (Wang et al., 2009, 2014; Lv et al., 2014; Shi et al., 2014). In the case of OsSPX-MFS proteins, OsSPX-MFS1 and OsSPX-MFS2 have been found to be involved in Pi transport and homeostasis of plants (Lin et al., 2010; Wang et al., 2012, 2015).

Tissue specific expression analysis revealed that the *OsNLA1* gene is mostly expressed in leaf and sheath of rice, followed by stem, inflorescence, and roots (**Figure 2**), which is somewhat different from Arabidopsis. The *AtNLA* expression was greatest in roots and stems, followed by leaves (Peng et al., 2007). This suggests that the spatial expression of NLA is regulated differently in monocots and dicots. The higher expression of *OsNLA1* gene in rice leaves and sheaths can be explained in terms of its importance in the function of translocation and remobilization of Pi between old and young leaves. Interestingly, within rice, SPX genes from different clades also exhibit differential tissue specific expression patterns. For example, members of the SPX-Major Facility Superfamily; SPX-MFS1-3 are mainly expressed in the shoot tissues compared to roots (Lin et al., 2010; Wang et al., 2012). However, members of the SPX subfamily, such as OsSPX1-3 are expressed differently, where higher transcript levels were found to be in roots, followed by leaves and callus compared to other tissues (Wang et al., 2014). Similarly, OsSPX6 is expressed more in roots and leaf tissues compared to other tissues and OsSPX5 is mainly expressed in root tissues (Wang et al., 2009). This suggests that Pi homeostasis in rice is regulated in a tissue dependent- manner with a different level of contribution by each subfamily of the SPX domain harboring proteins.

*OsPHO2*, another Pi utilization related gene, was also reported to have its highest expression in the sheath and the mature leaves (Cao et al., 2014). The similarity in the expression patterns of OsNLA1 and OsPHO2 suggested that there is a cooperative role in the regulation of Pi homeostasis in rice plants. PHO2 is an E2 enzyme (E2 conjugase protein) and NLA is an E3-ligase, and these two proteins have recently been shown to interact with each other in Arabidopsis as demonstrated using Y2H and *in vitro* pulldown assays, and this interaction was able to promote the autoubiquitination of NLA *in vitro* (Park et al., 2014). The detailed genetic and biochemical analyses further revealed that interaction between NLA and PHO2 is important in regulating Pi homeostasis by polyubiquitination and subsequent turnover of the phosphate transporter 2 (PT2) through the 26S proteasomal pathway in *A. thaliana* (Park et al., 2014). Apparently, OsNLA1 was also found to interact with OsPHO2 in our Y2H assay which suggests that OsNLA1 and OsPHO2 interaction may be a conserved phenomenon in monocots and dicots to maintain Pi homeostasis (**Figure 8**). However, during the reviewing process of our paper, another paper was published on the characterization of OsNLA1 in rice (Yue et al., 2017). This study also revealed the importance of OsNLA1 in the maintenance of Pi homeostasis in rice. Similar to our findings, *Osnla1* mutants exhibited chlorosis and toxicity symptoms on the leaf blades due to hyperaccumulation of Pi. However, contrary to our finding, OsNLA1 was not found to interact with PHO2 which led the authors to conclude that OsNLA1 degrade phosphate transporters (PT2 and PT8) possibly by interacting with other, yet unknown, E2 ligases to maintain the Pi homeostasis in rice (Yue et al., 2017). The apparent differences in the outcomes of interaction studies between OsNLA1 and OsPHO2 seem to result from the use of two different experimental approaches. We employed Y2H assay, whereas, Yue et al. (2017) used BIFC assay in tobacco leaves to study the interactions between OsNLA1 and OsPHO2. It is possible that OsNLA1 do interact with an E2-ligase/s other than PHO2 to degrade PT2 and PT8 in rice as concluded by Yue et al. (2017). However, it is important to mention that both BIFC and Y2H approaches may sometimes yield false negative or positive results. Therefore, it is necessary to confirm the results of such interactions by employing alternative approaches. The monitoring of degradation of PT2 and PT8 proteins in rice WT and *pho2* mutant backgrounds expressing inducible OsNLA1 constructs may provide confirmatory information if the degradation of PT2 and PT8 proteins by OsNLA1 requires OsPHO2 as demonstrated by Park et al. (2014) in *A. thaliana*.

OsNLA1 is a negative regulator for Pi/P accumulation in rice plants. This is supported by over-accumulation of Pi in OsNLA1 knockdown lines (**Figure 4**) through markedly greater Pi transport or uptake in roots, especially under low nitrate levels (**Figure 6**). This excessive Pi caused Pi toxicity in the leaf tissues of the knockdown plants but not in wild-type or in the over-expression lines. Similar Pi toxicity symptoms were also found in *Atnla* mutants under low nitrate and sufficient Pi conditions due to over-accumulation of Pi (Kant et al., 2011). Furthermore, the negative phenotypic effect of OsNLA1 knockdown lines was also obvious when grown under higher than optimal Pi (HP) as demonstrated by reduced plant height and intense leaf necrosis, especially at the tip of the leaf blade (**Figures 3E,F**). Interestingly, these toxicity symptoms were intensified when the OsNLA1 knockdown lines were grown in nutrient conditions with a combination of high Pi and low nitrate (**Figure 5**). This resulted in greater accumulation of Pi in OsNLA1 knockdown lines up to toxic levels on account of lack of function of OsNLA1, and these plants failed to maintain their Pi homeostasis. This is in agreement with the Arabidopsis *nla* mutants, which also over-accumulated Pi more than fivefold compared to the wild-type under low nitrate and high Pi levels (Kant et al., 2011). In contrast to OsNLA1 knockdown lines, OsNLA1 over-expression lines had significantly lower levels of Pi when grown under high Pi and low or sufficient nitrate (**Figure 5**) and sufficient Pi and low nitrate (**Figure 4**). This further supports the negative effect of OsNLA1 on Pi uptake and transport in rice.

We also investigated the effect of different levels of ammonium on Pi accumulation in the leaves since this is the most abundant nitrogen source in paddy fields (Yu, 1985; Tabuchi et al., 2007). However, there was no clear correlation between ammonium supply and Pi accumulation in rice plants, which was different from nitrate. This suggests the existence of a nitrate-specific cross-talk mechanism to regulate Pi accumulation in pant tissues. This is in agreement with previous reports in Arabidopsis and maize where reduced nitrate levels in the soil resulted in higher accumulation of Pi in the shoot tissues (Kant et al., 2011; Schlüter et al., 2012). Other studies have also revealed the significance of this antagonistic interactions between nitrate and Pi in the regulation of root development when Pi is limiting or deficient. For example, overexpression of miRNA444a in rice plants, an Pi-starvation-inducible gene, led to the activation of nitrate signaling pathway only under Pi-starvation conditions, which resulted in transgenic plants to produce longer primary roots with fewer lateral roots compared to wild-type (Yan et al., 2014). Similarly, the transcriptome analysis of *gpa1-5* mutant which exhibited several developmental phenotypes including the presence of longer roots with fewer lateral roots also revealed the induction of several nitrate starvation/assimilation genes such as *NRT2.1, ICDH*, and *ASN1* and reduction of phosphate use efficiency genes such as *WRKY75, PHT1*, and *LPR1* compared to wild-type (Chakraborty et al., 2015). Remarkably, the development of primary root under Pi deficient conditions was found to be largely dependent on the presence of nitrate in the growth medium, which is suppressed by nitrate-inducible HRS1 and HHO1 transcription factors (Medici et al., 2015). These observations are also in agreement with the study which investigated the impact of 32 binary combinations of nitrogen, phosphorus, potassium, sulfur, and light on the modulation of various root system architecture parameters in Arabidopsis (Kellermeier et al., 2014). The cross-talk/interactions between different nutrient signaling pathways thus appears to be a general mechanism which allows plants to respond to various developmental and environmental signals to regulate their growth (Kant et al., 2011; Kellermeier et al., 2014; Medici et al., 2015).

The addition of N and Pi fertilizers greatly enhances crop yields, but this comes at a high economic cost and leads to major environmental pollution problems. Usually, the availability of Pi in the soil is low due to its low solubility, low mobility and tightly bound nature to soil particles and also because of the nutrient imbalance due to the relatively large application of N fertilizers (Hinsinger et al., 2011). Therefore, the development of genetically improved crops for better nutrient use efficiency would be helpful for sustainable crop production. In this research, we have uncovered the critical importance of OsNAL1 on Pi homeostasis in rice, which was found to be dependent on nitrate but not ammonium levels in the soil. This finding can be used to identify crop cultivars with different levels of expression of OsNAL1to develop rice cultivars that can take up and accumulate enough Pi even under low Pi and nitrate levels in soil. This will also result in better utilization of Pi in balance with the soil nitrate supply.

## Author Contributions

SZ, KR, KM, Y-MB, and SR designed the experiments. SZ, KR, and KM performed the experiments and analyzed the data. Y-MB and SR supervised the experiments. SR conceived the project. All authors contributed to the writing.

## Conflict of Interest Statement

The authors declare that the research was conducted in the absence of any commercial or financial relationships that could be construed as a potential conflict of interest.

## Footnotes

1 http://rice.plantbiology.msu.edu/

2 http://www.megasoftware.net/index.php

3 https://tos.nias.affrc.go.jp/index.html.en

4 http://www.ncbi.nlm.nih.gov/BLAST

## Supplementary Material

FIGURE S1 Comparison of phenotype of OsNLA1 transgenic plants. **(A)** Plant phenotype and **(B)** shoot biomass content of wild-type, knockdown lines (*osnla1-1, osnla1-2*, and *osnla1-3*) and over-expression lines (OsNLA-OE1, OsNLA-OE2, and OsNLA-OE3) were grown with sufficient nutrients (including optimum nitrate and Pi levels) for 8 weeks in soil-pots. All genotypes showed the same phenotype at both growth stages (4 and 8 weeks after sowing). Data are means ± SD of ten to fifteen replicates (*n* = 10–15).

FIGURE S2 Analysis of total P accumulation in shoots and roots of OsNLA1 transgenic lines under sufficient and low nitrate levels. **(A)** Total P content of shoots and roots of wild-type, knockdown and over-expression lines, grown in hydroponics under sufficient (Control) and low nitrate levels for 4 weeks. Knockdown lines (*osnla1-1, osnla1-2*, and *osnla1-*3) had the greatest total P contents in shoots and roots under low nitrate (LN) level. The over-expression lines (OsNLA-OE1, OsNLA-OE2, and OsNLA-OE3) and wild-type had the same amount, except for roots grown in low nitrate. **(B)** The percent P increase by low nitrate treatment compared to the control. **(C)** The percent P change of knockdown and over-expression lines by low nitrate treatment, relative to the wild-type. Different letters indicate significant differences at *P* ≤ 0.05 level (ANOVA, LSD test). Comparison was done for shoots and roots separately. Data are means ± SD of three to five replicates (*n* = 3–5).

FIGURE S3 Relationship between applied ammonium in the medium and Pi accumulation in wild-type and *Osnla1-1* knockdown lines. Plants were grown in both hydroponics and soil, providing them with optimum Pi (300 μM and 1 mM, respectively) and different ammonium levels (in hydroponics, 300 μM being the optimum and 90 μM was lower than optimum; in soil, 10 mM being the optimum and 1 mM was sub-optimal/low). Low ammonium treatments did not result in accumulating higher Pi levels in the shoot of both genotypes. Different letters indicate a significant difference at *P* ≤ 0.05 level. Data are means ± SE of three to five replicates (*n* = 3–5).
