# Supplementary figures and images for: Altered Expression of OsNLA1 Modulates Pi Accumulation in Rice (Oryza sativa L.) Plants
Source: Front Plant Sci. 2017 Jun 2;8:928. doi: 10.3389/fpls.2017.00928 (PMC5454049; doi:10.3389/fpls.2017.00928)

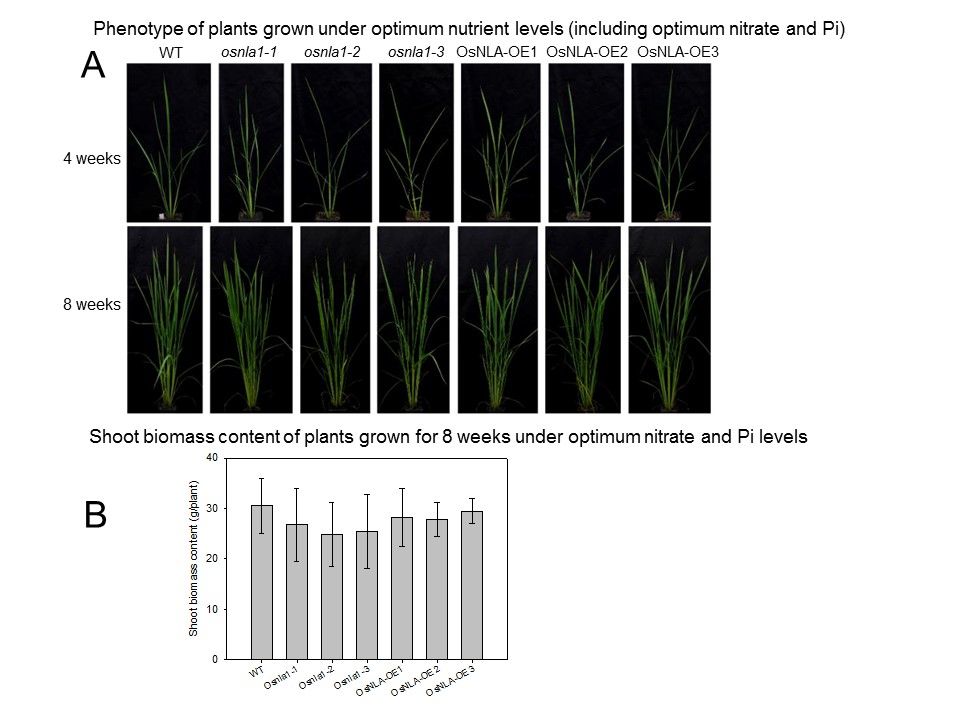

Supplement: FIGURE S1 — Comparison of phenotype of OsNLA1 transgenic plants. (A) Plant phenotype and (B) shoot biomass content of wild-type, knockdown lines (osnla1-1, osnla1-2, and osnla1-3) and over-expression lines (OsNLA-OE1, OsNLA-OE2, and OsNLA-OE3) were grown with sufficient nutrients (including optimum nitrate and Pi levels) for 8 weeks in soil-pots. All genotypes showed the same phenotype at both growth stages (4 and 8 weeks after sowing). Data are means ± SD of ten to fifteen replicates (n = 10–15). [file Image_1.jpg]

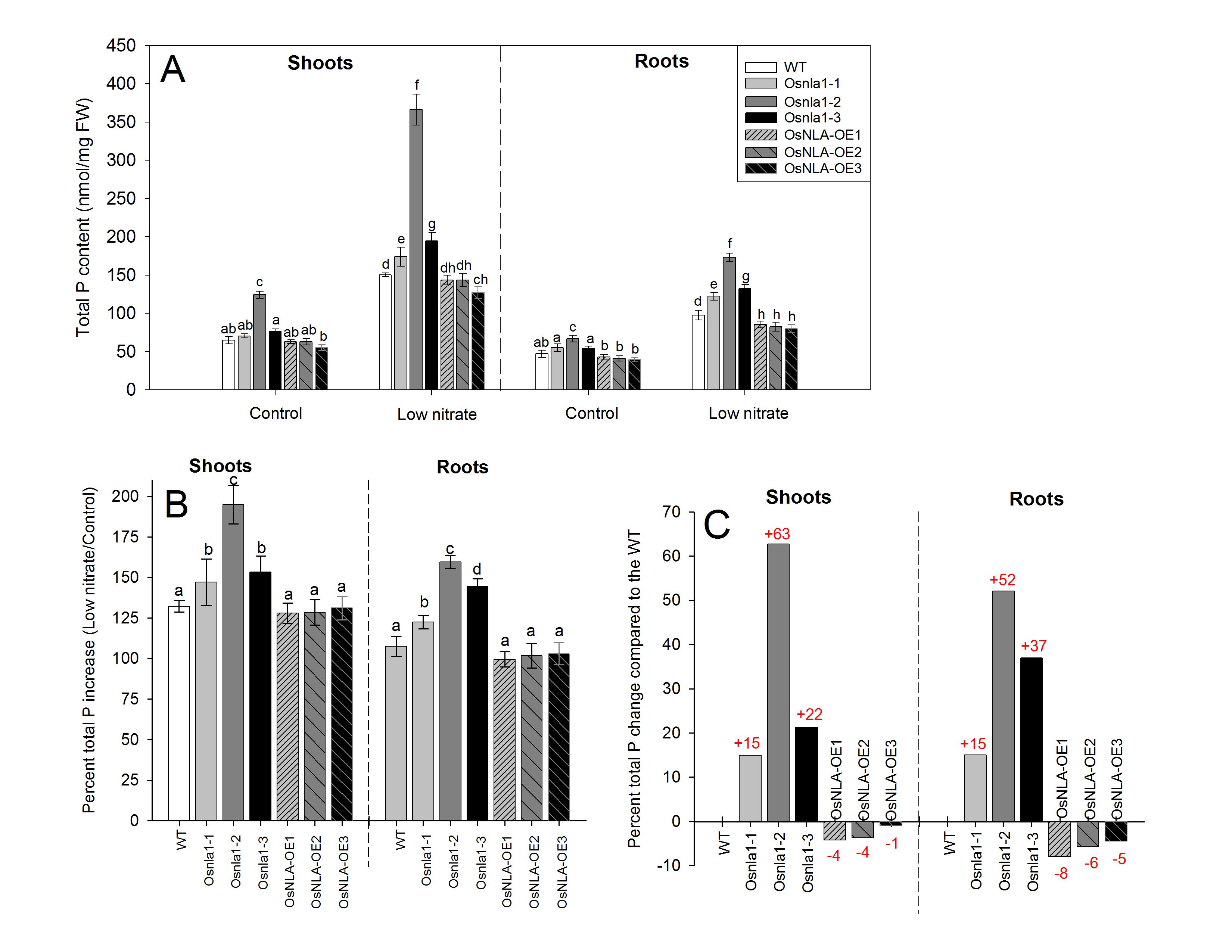

Supplement: FIGURE S2 — Analysis of total P accumulation in shoots and roots of OsNLA1 transgenic lines under sufficient and low nitrate levels. (A) Total P content of shoots and roots of wild-type, knockdown and over-expression lines, grown in hydroponics under sufficient (Control) and low nitrate levels for 4 weeks. Knockdown lines (osnla1-1, osnla1-2, and osnla1-3) had the greatest total P contents in shoots and roots under low nitrate (LN) level. The over-expression lines (OsNLA-OE1, OsNLA-OE2, and OsNLA-OE3) and wild-type had the same amount, except for roots grown in low nitrate. (B) The percent P increase by low nitrate treatment compared to the control. (C) The percent P change of knockdown and over-expression lines by low nitrate treatment, relative to the wild-type. Different letters indicate significant differences at P ≤ 0.05 level (ANOVA, LSD test). Comparison was done for shoots and roots separately. Data are means ± SD of three to five replicates (n = 3–5). [file Image_2.jpg]

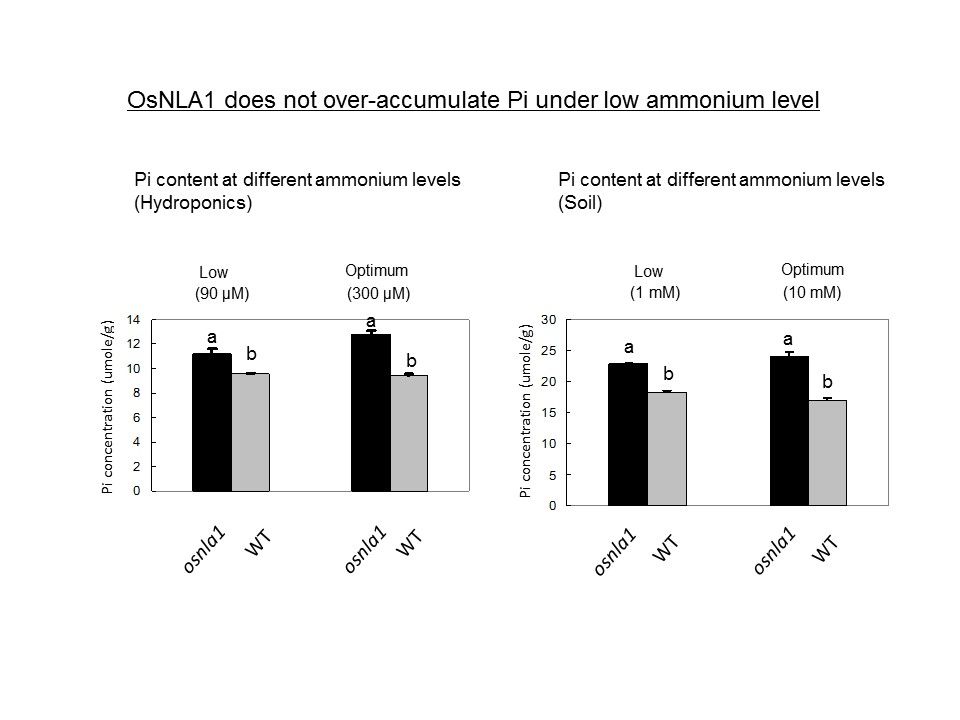

Supplement: FIGURE S3 — Relationship between applied ammonium in the medium and Pi accumulation in wild-type and Osnla1-1 knockdown lines. Plants were grown in both hydroponics and soil, providing them with optimum Pi (300 μM and 1 mM, respectively) and different ammonium levels (in hydroponics, 300 μM being the optimum and 90 μM was lower than optimum; in soil, 10 mM being the optimum and 1 mM was sub-optimal/low). Low ammonium treatments did not result in accumulating higher Pi levels in the shoot of both genotypes. Different letters indicate a significant difference at P ≤ 0.05 level. Data are means ± SE of three to five replicates (n = 3–5). [file Image_3.jpg]
