# Supplementary material for: Altered Expression of OsNLA1 Modulates Pi Accumulation in Rice (Oryza sativa L.) Plants
Source: Front Plant Sci. 2017 Jun 2;8:928. doi: 10.3389/fpls.2017.00928 (PMC5454049; doi:10.3389/fpls.2017.00928)
Supplement: Supplementary file 4 [file Table_1.docx]

**Suppl Table S1** Sequences of primers used for qRT-PCR

| **Gene ID** | **Forward primer (5′ → 3′)** | **Reverse primer (5′ → 3′)** |
| --- | --- | --- |
| yOsNLA1  yOsPHO2 | GGACATATGAAGTTTGCCAAGA  TCGAATTCATGGATCTATATGC | CCAACGGATCCATCACATGCCCA  ATGCTCGAGGTCACGGGCTGCAG |
